# Introduction of SARS in France, March–April, 2003

**DOI:** 10.3201/eid1002.030351

**Published:** 2004-02

**Authors:** Jean-Claude Desenclos, Sylvie van der Werf, Isabelle Bonmarin, Daniel Levy-Bruhl, Yazdan Yazdanpanah, Bruno Hoen, Julien Emmanuelli, Olivier Lesens, Michel Dupon, François Natali, Christian Michelet, Jacques Reynes, Benoit Guery, Christine Larsen, Caroline Semaille, Yves Mouton, Daniel Christmann, Michel André, Nicolas Escriou, Anna Burguière, Jean-Claude Manuguerra, Bruno Coignard, Agnés Lepoutre, Christine Meffre, Dounia Bitar, Bénédicte Decludt, Isabelle Capek, Denise Antona, Didier Che, Magid Herida, Andréa Infuso, Christine Saura, Gilles Brücker, Bruno Hubert, Dominique LeGoff, Suzanne Scheidegger

**Affiliations:** * Institut de Veille Sanitaire, France;; † Institut Pasteur, France; ‡ Centre Hospitalier de Tourcoing, France; § Centre Hospitalier Universitaire (CHU) de Besançon, Besançon, France; ¶ CHU de Strasbourg, Strasbourg, France; # CHU de Bordeaux, Bordeaux, France; ** Hôpital d’Instruction des Armées, Brest, France; †† CHU de Rennes, Rennes, France; ‡‡ CHU de Montpellier, Montpellier, France; §§ Cellule Inter-Régionale d’Epidémiologie Ouest, Rennes, France; ¶¶ Direction Départementale de l’Action Sanitaire et Sociale *(*DDASS) du Finistére, Rennes, France; ## DDASS du Doubs, Besançon, France

**Keywords:** severe acute respiratory syndrome, epidemiology, transmission, coronavirus, commercial flight, research

## Abstract

We describe severe acute respiratory syndrome (SARS) in France. Patients meeting the World Health Organization definition of a suspected case underwent a clinical, radiologic, and biologic assessment at the closest university-affiliated infectious disease ward. Suspected cases were immediately reported to the Institut de Veille Sanitaire. Probable case-patients were isolated, their contacts quarantined at home, and were followed for 10 days after exposure. Five probable cases occurred from March through April 2003; four were confirmed as SARS coronavirus by reverse transcription–polymerase chain reaction, serologic testing, or both. The index case-patient (patient A), who had worked in the French hospital of Hanoi, Vietnam, was the most probable source of transmission for the three other confirmed cases; two had been exposed to patient A while on the Hanoi-Paris flight of March 22–23. Timely detection, isolation of probable cases, and quarantine of their contacts appear to have been effective in preventing the secondary spread of SARS in France.

Severe acute respiratory syndrome (SARS) was recently identified as a new clinical entity (*1*). SARS likely originated in the Guangdong Province of People’s Republic of China (*2*) and subsequently spread worldwide as infected persons traveled. During the 2003 outbreak, SARS was primarily transmitted by person-to-person contact between healthcare workers or household members and ill patients (*2*). Community transmission also occurred in several of the most affected areas, and an explosive outbreak from a common source occurred in Amoy Garden in Hong-Kong (*3*). As of June 2003, a total of 8,477 probable cases and 811 deaths had been reported from 32 countries (*4*). A novel coronavirus has been identified as the cause of SARS (*5*–*7*). Based on current knowledge, SARS is transmitted from symptomatic patients by close direct or indirect contacts through respiratory droplet secretions (*2*). In specific situations, other modes of transmission, such as airborne spread, may be possible (*8*). The incubation period ranges from 2 to 10 days, allowing SARS to spread over long distances by infected persons who travel (*8*,*9*).

We describe how SARS was introduced in France through a single patient who returned from Vietnam on March 23 and present data that suggest transmission from this patient to other passengers may have occurred during his flight back from Hanoi to Paris.

## Material and Methods

After the World Health Organization (WHO) alert on March 12, 2003, a centralized surveillance system was set up for SARS in France (*10*). All persons who returned from an area affected by recent transmission, had been in contact with a probable case during the previous 10 days, and in whom fever was >38°C, with cough or difficult breathing, were advised to call the emergency service. These persons were transported to the closest university-affiliated infectious disease ward or one of the nine infectious disease wards designated as a regional reference center in the French plan of action against bioterrorism, using masks for droplet protection. After performing clinical and biologic evaluation and chest x-ray, the attending clinician notified the Institut de Veille Sanitaire through a unique telephone number. On the basis of the results of the initial and subsequent evaluations, each notified case was either discharged, kept as a suspect case, or classified as a probable case using the WHO SARS case definition (*10*,*11*). Probable and suspected case-patients were kept in isolation until recovery or until the diagnosis was changed, respectively. For this investigation, a probable case of SARS was defined as previously described (*12*).

For patients who fulfilled the definition of a probable case, respiratory secretion specimens were taken from the nose, throat, or sputum to detect for SARS–associated coronovirus (CoV) by reverse transcription–polymerase chain reaction (RT-PCR) (*7*) at the National Reference Center for Influenza (Northern France), Institut Pasteur, Paris. RNA extraction and RT-PCR mixes were prepared in designated rooms. RT-PCR procedures included appropriate negative and positive controls in each run: two negative controls for the extraction procedure and one water control and one positive control for each PCR run. Two RT-PCR, either both nested or one nested and one real-time, were performed for each sample. Real-time RT-PCR, using the SARS-CoV detection kit from Artus (Germany), included an internal control that detected PCR inhibitory substances. One-step nested RT-PCR targeting either the Bernahard Nocht Institute (BNI) or the Centers for Disease Control and Prevention (CDC) fragment of the polymerase gene was used (*7*,*13*). When real-time RT-PCR was performed, which targets the BNI fragment, the other RT-PCR was the nested RT-PCR targeting the CDC fragment of the polymerase gene. The real-time and nested RT-PCR, which targeted the BNI fragment reliably, detected 10 copies of RNA in the assay corresponding to 800 RNA molecules per milliliter of specimen.

Acute and convalescent serum samples were also obtained from probable cases. They were tested for immunoglobulin (Ig) G antibodies against the SARS-CoV using indirect immunofluorescence with Vero E6 cells infected by the SARS-CoV, negative control Vero E6 cells and fluorescein-labeled goat antihuman IgG. Results of serologic testing were considered positive either in case of seroconversion or a fourfold increase of observed titers, or if the serum exhibited a titer >160. The detection limit of our indirect immunofluorescence assay corresponded to the first dilution used: 1/40.

For each probable and confirmed case, information was collected on clinical symptoms, chest x-ray findings, leukocyte counts, illness onset date, demography, all possible contacts with a probable case, and exposures when traveling to affected area (contact with any hospital or place of potential transmission). Persons who did not use masks for droplet protection and had contact with a symptomatic probable or confirmed case of SARS were quarantined at home for 10 days after exposure and contacted daily by telephone. As recommended by WHO, this follow-up included the passengers who sat within two rows of a SARS case-patient on the Air France Hanoi-Paris flight of March 22 and 23, 2003 (*14*). The crew of the Air France flight was also followed for 10 days by the Air France medical service. During follow-up interviews with the passengers seated close to the index patient (patient A), we obtained a detailed description of his clinical condition, his movements in the aircraft, the contacts he may have had with other persons on board, and the timing of his boarding and deplaning in relation to other passengers, including the stopover in Bangkok. Passengers on a flight in which a person with a symptomatic probable case had traveled were informed publicly through the media and mail of the potential exposure and advised to call the emergency service phone number to be evaluated and admitted to the closest university-affiliated infectious disease ward if a fever of >38°C developed within 10 days of the flight.

We estimated the incidence density of SARS among passengers who sat within two rows of a case of SARS in the AF171 flight of March 22–23 by using the total number of person-hours as the denominator. Ninety-five percent confidence intervals (95% CI) were calculated by using the exact binomial method (*15*).

## Results

As of April 30, a total of 394 suspected cases had been notified to the Institut de Veille Sanitaire and 5 (1.3%) met the definition of a probable case of SARS. Four were men, and their ages were 26 to 56 years. All had fever >38°C, four with nonproductive cough and two with dyspnea. None had diarrhea. Chest x-rays showed interstitial pneumonia in four patients (bilateral for three) and alveolar consolidation in one. Lymphocyte counts were 170 to 1,400/mm^3^. Four patients were lymphopenic (<1,000/mm^3^); the same four patients also had thrombocytopenia. Severe hypoxemia that required mechanical ventilation developed in one patient (the index case, patient A). Four patients had been discharged from the hospital within 8 to 21 days after onset, and one died (patient A) from intensive-care complications 95 days after admission.

**Figure 1:**
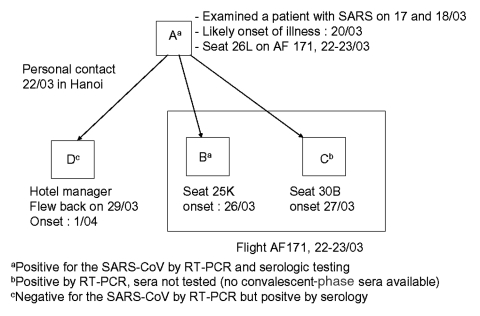
Cases of SARS, by date of onset and exposure, laboratory results and type of exposures, France, March-April, 2003.

RT-PCR was positive for SARS-CoV in at least three of the respiratory secretion samples taken on at least 2 different days after onset of symptoms for three of the five patients. Acute and convalescent serum samples were obtained for four of the probable cases, and seroconversion to SARS-CoV occurred in three samples, including samples from the patient for whom RT-PCR was negative (patient D, Figure 1). However, for patient D, the only respiratory samples available for RT-PCR were taken on day 2 after onset.

Our subsequent analysis is restricted to the four confirmed cases (case A to D, Figure 1). All four cases were related to the outbreak that occurred in the French Hospital in Hanoi, Vietnam (*2*). The index case (patient A), who had worked in this hospital, was the most probable source of secondary transmission to the other three cases. On the basis of information obtained from his colleagues, on March 16 and 17, he was known to have examined, without respiratory protection from droplet secretions, an ill physician in whom SARS subsequently developed. Although no precise date of onset is available for patient A, interviews with persons he had met in Hanoi during the few days before his departure indicate that symptoms, such as cough and severe fatigue, had developed as early as March 20.

From March 26 to April 1, three secondary cases occurred (Figure 1), with incubation periods of 3, 4, and 10 days. Two cases occurred among the 371 passengers (166 boarded in Hanoi of whom 5 left in Bangkok, and 205 boarded in Bangkok) and 30 flight attendants of the Air France Hanoi-Bangkok-Paris flight of March 22–23. The last case (case D) was the manager of the hotel where patient A stayed in Hanoi. He became ill on April 1, a total of 3 days after returning to Vietnam on March 29 through another flight. He had had close contact with patient A on March 22 while greeting and giving him his mail before departure (Figure 1). No other exposure to cases of probable SARS or places where transmission of SARS had occurred in Hanoi could be documented for patient D within 10 days of symptom onset.

**Figure 2:**
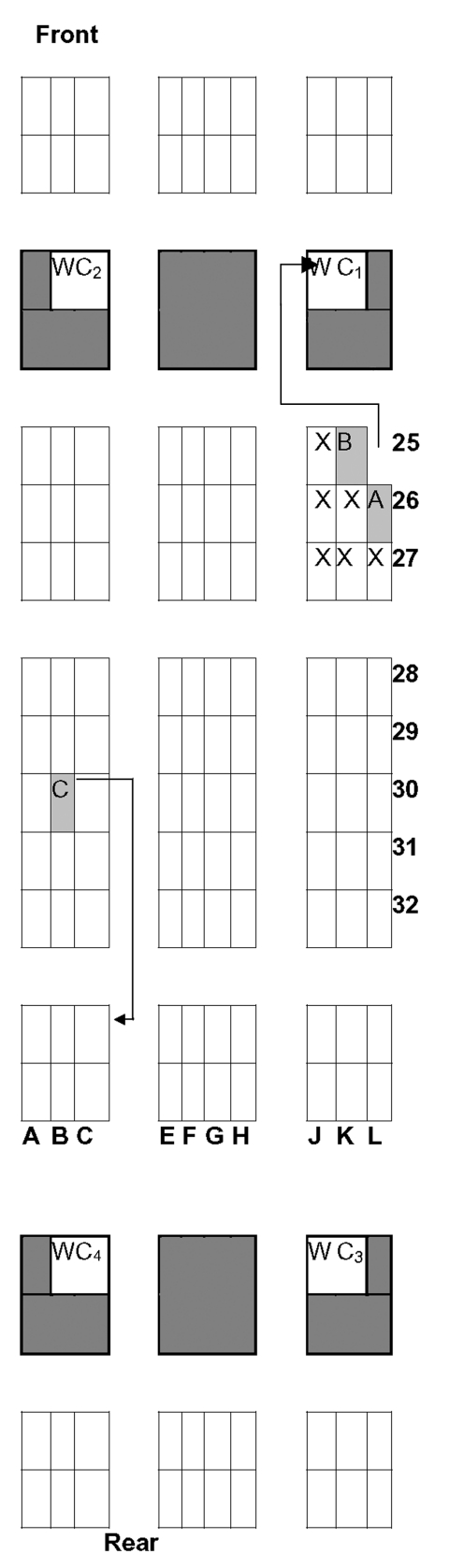
Seats occupied by Probable case of SARS and close contacts to patient A, Air France Flight 171, Hanoi-Paris, 22-23 March, 2003. Numbers and letters in bold indicate seat lanes and rows, respectively. Patient A occupied seat 26L (next to the window). Seats of close passengers who were followed for 10 days are indicated by an X. They included two passengers who sat in the row ahead (25K and 25J, there was no seat at 25L), two passengers who occupied seats 26K and 26J, and three passengers who sat in the row behind (27J, 27K and 27L). A row with no seats separated row 27 from row 28; a partition separated row 25 from the rest of the cabin. Consequently, passengers seated in rows 28 and 24 were excluded. The lavatories are indicated (WC). Patient A and B used the front lavatory (WC1) while patient C used the one in the back (WC4). The arrow between seat 26L and the lavatory WC1 indicates that patient A passed through the empty space between the plane wall and seat 25K where patient B was seated.

Seven persons sat within two rows of patient A during the AF 171 flight (Figure 2), two of whom were medical doctors and did not know him. They indicated that patient A was breathing rapidly (superficial polypnea) and exhibited extreme pallor and pursed lips during the entire flight. He remained calm, had no cough, and left his seat at least twice between Bangkok and Paris to go to the front lavatory; at each move, he passed through the space between the plane wall and seat 25K (Figure 2). During the stopover in Bangkok, he disembarked with the passengers on the flight from Hanoi to Bangkok and then reboarded the plane before the passengers who embarked in Bangkok. On landing at Charles de Gaulle (CDG) Paris Airport, he disembarked among the last passengers (about 20 passengers left the plane after him) and was cared for by the CDG medical services along with two other physicians who had worked in the French Hospital in Hanoi and were on the same plane.

Of the seven passengers who sat within two rows of case A, one developed SARS (case B, seat 25K), which accounted for an incidence density rate of 1 per 100 person hours of exposure (1/98 hours; 95% CI 0.02 to 5.4). He reported having handled the same aircraft magazines and using the same lavatory as patient A (WC1, Figure 2). Within 10 days of onset and while in Hanoi, case B did not report any contact with the French hospital, other hospitals, or with any SARS patients, nor did he stay at the same hotel as patient A. Another passenger who sat near patient A (26K) reported a sore throat and a temperature of 37.6°C once during follow-up.

The second case (case C) sat in seat 30B. He boarded the plane in Bangkok and did not know patient A and did not recall having had any interaction with him during the flight. He used the toilets to the rear behind his seat while patient A used the toilets nearest his seat up front (Figure 2). He was among the first passengers leaving the plane. He did not report any contact with ill persons or hospitals while in Thailand.

Other contacts of patient A included two persons who shared the same car to the Hanoi airport, one of whom had met him for 2 hours before departing; two physicians who had worked in the Hanoi French hospital and left the plane with him; and four healthcare workers of CDG medical services who cared for him. Two taxi drivers (one 1 1/2-hour drive from CDG to his home and one 1/2-hour drive from his home to the infectious disease hospital where he was admitted) were also exposed to patient A, who was then wearing a mask. None of these nine persons had any symptoms during the 10 days after exposure.

SARS did not develop in any of the 30 unprotected persons who had contact with the three secondary confirmed cases after their onset of fever (duration of contact <1/2 hour to 3 days; <2 hours for 26 [86.7%]). However, a febrile illness for 2 days, with no other symptoms, developed in a household contact of patient D, who had a close unprotected contact with him for about 1/2 hour at onset of his symptoms (malaise and fever); a chest x-ray was normal and lymphocyte count was 441/mm^3^. RT-PCR on nasal and pharyngeal swab was negative for SARS-CoV. Three healthcare workers who cared for patient D and used masks for droplet protection had brief episodes (<24 hours) of mild fever without any respiratory symptoms and chest x-ray changes. These three episodes were attributed to a common, unidentified, local viral infection.

## Discussion

The surveillance system was able to detect the first patient with SARS (patient A) and one of his secondary case-patients (patient D). Follow-up of passengers seated within two rows of patient A, and the information given to the other passengers of flight AF171 flight allowed patients B and C to be identified. Therefore, all case-patients were identified early in the course of the disease and placed under isolation, which contributed to reduction in the risk of secondary transmission and diffusion (*16*). Only four of the five probable cases were confirmed either by RT-PCR or serologic testing, although all five met the probable SARS case definition. Although specific, the sensitivity of the RT-PCR–based detection technique remains to be fully evaluated (*7*). In addition, the time at which respiratory specimens were taken could account for the fact that virus shedding remained undetected for one patient (patient D).

Of the persons who came into contact with a symptomatic SARS patient in France, 30 did not have masks for droplet protection and were exposed, and 26 (86.7%) were exposed for a limited amount of time at the onset of illness. No probable case of SARS was identified among these persons; a household contact of patient D had a febrile illness (>38°C) without any other symptoms and tested negative for the SARS-CoV by RT-PCR. Four contacts of SARS cases had an episode of transient, mild or low-grade fever without other signs, including three healthcare workers of the hospital where patient D had been admitted and the passenger seated next to patient A during the AF171 flight. Specific antibody testing will be the only way to evaluate if these persons with mild symptoms could have been infected by the SARS-CoV.

Since no other exposure could be found within 10 days of onset for cases B and C, their probable source of infection is contact with patient A while in flight, boarding, or disembarking flight AF 171. For patient B, we cannot formally exclude an unrecognized community exposure in Hanoi during the 10 days before departure. However, the fact that the SARS outbreak was controlled quite rapidly (*17*), without any formal documentation of community transmission, a large unrecognized community transmission most likely did not occur. Patient B, in addition to sitting within two rows of patient A, had contact with patient A when he moved to and from the lavatory (at least four close contacts while going and coming at least twice from the lavatory). Although a precise date of fever onset is not available for patient A, it appears that he was already symptomatic in the plane and was likely infectious. This finding is based on the following evidence: 1) some persons who had met him in Hanoi before his departure reported that he had fatigue and fits of cough; 2) the passengers closest to him on the plane reported that he was dyspneic; and 3) his initial evaluation at admission to hospital on March 23 showed bilateral extended interstitial pneumonia and hypoxemia. The last strongly supports the hypothesis that his illness was ongoing for 3 to 8 days (*1*,*5*,*8*).

For patient C, the exact mode of acquisition of SARS remains a matter of debate, since he was neither found to have close contact with patient A nor other documented exposure. He had been traveling to Thailand, a country where local transmission has never been reported by WHO (*18*). Although airborne transmission on the plane cannot be ruled out, a possible hypothesis is an undocumented direct or indirect contact with patient A while boarding or on the plane. Our investigation also indicates that the risk for acquiring SARS after a contact with a symptomatic case is very heterogeneous, since prolonged contact does not necessarily result in transmission and, conversely, a brief or distant exposure might be sufficient. Factors that may explain this observation are the following: 1) the virus excretion varies over time, 2) the susceptibility to the SARS-CoV may vary among persons exposed, and 3) exposure results in asymptomatic infection.

Although our study is descriptive and was not designed to evaluate SARS control measures, our results support the usefulness of recommendations made to prevent the propagation of SARS through air travel (i.e., that persons suspected to have SARS should not fly [*14*]). We also believe that timely and sensitive surveillance associated with prompt and strict isolation of cases and quarantine of contacts were effective public health tools to limit the secondary spread of SARS in France.
